# A Systematic Review of Empirical Data from Behavior Analysis on Anti-Black Bias

**DOI:** 10.1007/s40614-026-00506-3

**Published:** 2026-06-09

**Authors:** Denise Aparecida Passarelli, Táhcita Medrado Mizael, Júlio C. de Rose

**Affiliations:** 1 Department of Psychology, Federal University of São Carlos, São Carlos, São Paulo Brazil; 2 Department of Clinical Psychology, University of Edinburgh, Edinburgh, UK

**Keywords:** Anti-Black bias, Behavior analysis, Implicit measures, Relational training

## Abstract

Despite growing attention to diversity, equity and inclusion (DEI), empirical research on anti-Black bias within behavior analysis remains scarce. This systematic review followed PRISMA guidelines and adopted a methodological and conceptual framework grounded in behavior analysis to identify and examine 23 empirical studies published in 27 behavior-analytic journals between 1964 and 2026. The selected studies were categorized into three methodological domains: (1) cross-sectional and correlational surveys; (2) validity of implicit and explicit measures for detecting anti-Black bias; and (3) experimental interventions on anti-Black bias. Cross-sectional and correlational studies examined associations between values, psychological flexibility, mental health, and racial attitudes, highlighting contextual influences on bias. Measurement studies primarily used relational frame theory–based tools, including the implicit relational assessment procedure (IRAP) and the Function Acquisition Speed Test (FAST), demonstrating sensitivity to implicit anti-Black biases not captured by self-report measures. Intervention studies included equivalence-based training, particularly with children, showing promising but preliminary effects in modifying biased relational networks. Interventions with adults also yielded favorable outcomes, especially those involving values clarification and exposure to relationally inconsistent stimuli. Across cross-sectional studies, common limitations included reliance on convenience samples, incomplete reporting of sampling procedures, and inadequate control of confounding variables. Experimental studies frequently lacked control groups or baseline comparability included limited follow-up assessments, and relied on self-report or emerging measures with limited validation. Overall, findings highlight the need for a more cumulative, conceptually precise, and functionally grounded research agenda to advance antiracist efforts within behavior analysis.

**Supplementary Information:**

The online version contains supplementary material available at https://doi.org/10.1007/s40614-026-00506-3.

Racial bias, particularly anti-Black bias, remains one of society’s most persistent and socially damaging phenomena. Although psychology has studied racial bias extensively since the mid-20th century, behavior analysis has only recently begun to engage with this critical issue. Foundational studies such as Clark and Clark’s (1947) doll experiments demonstrated how racial segregation distorted Black children’s self-perception, laying the groundwork for research on racial attitudes. Developmental research has further demonstrated that racial attitudes emerge early and are shaped by social learning. For instance, Aboud et al. (2003) demonstrated that racial bias in children is largely driven by in-group favoritism rather than explicit out-group hostility, a pattern that can nonetheless contribute to the exclusion of peers from other racial groups.

In the early 2000s, psychology advanced theoretical models of implicit bias, particularly through evaluative conditioning paradigms (De Houwer et al., 2001; Hofmann et al., 2010). These models demonstrated how attitudes, especially racial attitudes, can be shaped by repeated pairings of social stimuli (e.g., Black faces) with positively or negatively valenced stimuli, even in the absence of explicit verbal reports describing the relevant stimulus–stimulus pairings. Evaluative conditioning research has shown how such pairings can both establish and modify racial evaluations (Kurdi & Banaji, 2017; Olson & Fazio, 2001, 2006; Zanon et al., 2014), highlighting the automatic, context-sensitive, and modifiable nature of racial evaluations.

Despite this expanding knowledge base, behavior analysis historically remained disconnected from this literature, due to its emphasis on directly observable behavior and its rejection of mentalistic constructs such as “attitudes” (Baer et al., 1968; Skinner, 1953). Although methodologically grounded, this hesitation also reflected the difficulty of defining complex phenomena like racism in functional terms and isolating their controlling variables in experimental contexts (Passarelli et al., 2023).

This historical gap began to close with theoretical developments within behavior analysis itself. Stimulus equivalence (Sidman, 1994) and, later, relational frame theory (RFT; Hayes et al., 2001) offered tools for analyzing racial bias as patterns of learned verbal relations and derived stimulus functions (de Carvalho & de Rose, 2014; Mizael & de Rose, 2017). These frameworks allowed behavior analysts to approach bias without abandoning a functional-analytic framework.

## Behavioral Definitions of Racial Bias

As mentioned, theoretical advances in behavior analysis provided an alternative to the mentalistic explanations often found in social psychology (Watters et al., 2023). Rather than attributing racial bias to internal states or cognitive beliefs, behavior analysis explains it in terms of functional relations among stimuli (Passarelli, Mizael et al., 2024a, Passarelli, Roche et al., 2024b; Mizael & de Rose, 2017) and the impact of contextual (Guerin, 2003) and cultural contingencies on behavior (Saini & Vance, 2021). Thus, from a behavioral perspective, racial bias can be defined as a pattern of relational responding in which individuals respond more favorably (or positively) or unfavorably (or negatively) toward stimuli related with racial categories (see Mizael & de Rose, 2017; see also Passarelli et al., 2023, Passarelli, Mizael et al., 2024a). These responses are shaped by both direct reinforcement histories and derived relational responding, often involving relational frames such as equivalence (e.g., “Black” = “bad”), comparison (e.g., “White is better than Black”), and/or opposition (e.g., “If white is good, then Black is bad”).

It is important to note that stimuli related to racial groups, such as skin tone, names, or facial features, can acquire evaluative functions (e.g., “good” or “bad”) through socially mediated learning processes. These derived relational responses may influence behavior in significant ways, guiding actions such as approach or avoidance (Lindström et al., 2014), preferences (Dehon et al., 2017; Torrez et al., 2024), or discriminatory behavior (Carvalho et al., 2022), even in the absence of direct experience. Racial bias, therefore, encompasses not only evaluative judgments but also observable patterns of preferential behavior (e.g., a preference to promote a white candidate over a Black candidate with an identical CV) and discriminatory actions (e.g., shooting an unarmed Black individual while refraining from shooting an armed white individual).

However, racial bias is neither linear nor uniform. Although negative evaluations may increase the probability of reduced preference and discriminatory behavior, these effects are strongly context dependent. In many situations, stimuli evaluated as negative (e.g., “Black” paired with “dangerous” or “unintelligent”) are less preferred in selection contexts (e.g., school admissions, workplace promotions) or more frequently targeted by exclusionary practices (e.g., disproportionate police stop). At the same time, those stimuli can also evoke topographically positive evaluations (e.g., “Black people are strong”). However, such positive-looking evaluations may function to maintain inequities by restricting access to roles associated with intellectual authority or decision making (e.g., executive or managerial positions), while positioning Black individuals as “more suitable” for physical or athletic activities (Ferrucci et al., 2013; Carton & Rosette, 2011).

Furthermore, positively evaluated stimuli are not always preferred. Women, for instance, are often positively evaluated (see Roche et al., 2025) and linked to traits such as warmth or nurturance (see Cartwright et al., 2016) yet may be less preferred in contexts where historically masculine functions (e.g., dominance, assertiveness, authority) are reinforced, such as leadership or power positions. This complexity highlights the importance of analyzing bias in terms of stimulus histories, contextual functions, and the contingencies that shape when, where, and how discriminatory patterns emerge.

### Explicit and Implicit Racial Bias

Racial bias can emerge from both implicit and explicit relational responses. From a functional standpoint, this distinction does not concern awareness or intentionality, but rather whether the behavior is controlled directly by environmental contingencies or indirectly through previously learned relational networks (De Houwer & Moors, 2012) and the contextual variables influencing behavior (Passarelli, Mizael et al., 2024a; Watters et al., 2023). Explicit bias involves verbal or overt responses that typically occur when individuals have sufficient time to respond according to socially mediated contingencies. These are commonly assessed through self-report instruments, such as Likert-type scales or structured interviews. In contrast, implicit bias consists of indirect, often rapid relational responses that emerge under time pressure or in ambiguous contexts (Barnes-Holmes et al., 2010a, b; Passarelli, Mizael et al., 2024a; Watters et al., 2023). These responses are shaped by long-standing relational histories and maintained by broader cultural contingencies, rather than immediate social feedback.

Over the past decades, computerized time-pressure procedures such as the implicit relational assessment procedure (IRAP; Barnes-Holmes et al., 2006) and the Function Acquisition Speed Test (FAST; O'Reilly et al., 2012) have been developed to assess rapid, contextually controlled relational responding. Both tasks rely on brief response windows to reduce the influence of self-presentation strategies and to reveal evaluative patterns shaped by participants’ learning histories.

The IRAP has been widely applied to racial bias research as a latency-based measure of derived relational responses (Barnes-Holmes et al., 2010a, b; Drake et al., 2015; Hussey et al., 2015; Power et al., 2017a, b). In Race-IRAP, participants complete blocks that either align with or contradict culturally dominant relational networks. In consistent blocks, selecting “True” to Black-negative or white-positive pairings is reinforced; in inconsistent blocks, this contingency is reversed. The primary index, the d-IRAP score, standardizes latency differences between these blocks. Positive D-IRAP values indicate relatively faster responding in blocks consistent with participants’ pre-experimental relational histories compared to inconsistent blocks, with the magnitude of the D-score reflecting the strength of this bias. For example, Hussey et al. (2015) reported significant d-IRAP scores indicating racial bias even among participants who self-reported egalitarian attitudes. This demonstrates that temporal constraints can reveal relational patterns that do not usually emerge under conditions that permit self-presentation or reflective control (for a review of IRAP studies about racial biases, see Mizael & de Almeida, 2019).

Likewise, the FAST has been used to examine evaluative racial bias in recent studies (Passarelli, Mizael et al., 2024a, Passarelli, Roche et al., 2024b; Roche et al., 2025; Rodrigues et al., 2022). The FAST measures how quickly participants learn arbitrary stimulus–response relations under time pressure. Participants are exposed to two types of blocks: in the consistent block, responses align with culturally dominant or experimentally established relations, whereas in inconsistent blocks, the relational contingencies are reversed. Differences in response latency between consistent and inconsistent blocks reflect the extent to which responding is influenced by participants’ learning histories and current contextual control. Rodrigues et al. (2022), for instance, employed the FAST to examine evaluative racial bias within verbal relational networks among university students. The procedure consisted of two blocks: one stereotype-consistent and one stereotype-inconsistent. In the consistent block, participants pressed “Z” for stimuli containing Black faces and negative words and pressed “M” for white and positive words. In the inconsistent block, these contingencies were reversed. Participants responded faster and more accurately in stereotype-consistent trials, and these FAST effects did not correspond to explicit self-reports. This dissociation suggests that speeded relational tasks can effectively capture brief relational responses that are less influenced by socially mediated reporting strategies.

Overall, implicit evaluations are themselves behavioral phenomena (De Houwer, 2019), measured by latency-based tasks that capture learned patterns of relational responding. These patterns have been consistently observed under controlled laboratory conditions (Barnes-Holmes et al., 2010a, 2010b; Cartwright et al., 2016; Passarelli, Mizael et al., 2024a, Passarelli, Roche et al., 2024b; Roche et al., 2025) and may extend to natural environments, particularly in situations involving time pressure, ambiguity, or weak social monitoring, where they may influence stimulus control, avoidance, and decision making, even when explicit reports suggest egalitarian verbal responses (Dehon et al., 2017; Lindström et al., 2014).

It is important to note, however, that the relationship between implicit measures and overt discriminatory behavior is not straightforward. Evidence from research using the implicit association test (IAT) suggests that latency-based tasks show modest predictive validity for real-world discrimination (Oswald et al., 2013; Larsson Taghizadeh, 2022), although even small effects may carry meaningful societal consequences (Gawronski et al., 2020; Greenwald et al., 2015, 2022; cf. Oswald et al., 2015). Predictive power is also influenced by methodological factors, including the degree of correspondence between the implicit measure and the behavioral outcome being predicted (Kurdi et al., 2019). Thus, implicit bias is best understood not as a hidden mental construct but as a history-shaped pattern of relational responding that may or may not influence real-life discriminatory outcomes. It is important to note that recent evidence suggests that such responding is contextually sensitive, with configurational variables modulating the expression of relational patterns captured by latency-based measures (Passarelli et al., 2026).

### The Current Study

This systematic review arrives at this critical juncture to synthesize empirical findings on anti-Black racial bias within behavior-analytic research. We examined how behavior analysis understands, measures, and intervenes upon anti-Black bias through its unique theoretical lens. By connecting behavior analysis’ historical foundations to its emerging role in bias research, this review aims to support the advancement of an empirically grounded, socially responsive behavioral science capable of addressing racial injustice.

Although prior work has addressed issues of racial bias within behavior analysis, including conceptual and narrative reviews (e.g., Matsuda et al., 2020; Mizael & de Rose, 2017; see also recent contributions such as Passarelli et al., 2023, and Zuch et al., 2024), these efforts have been primarily theoretical in nature. The present review extends this literature by adopting a systematic approach to the identification, appraisal, and synthesis of empirical studies, explicitly restricting inclusion to empirical work. In doing so, it prioritizes the organization of existing data and highlights the importance of ensuring that theoretical interpretations do not precede or outpace the available evidence. The review is specifically focused on anti-Black bias, recognizing that broader constructs such as racism are multifaceted phenomena that cannot be fully captured within a single analytic framework.

By narrowing the scope, this review provides a more precise characterization of the empirical evidence related to anti-Black bias within behavior analysis, identifies methodological patterns and limitations, and clarifies the extent to which current findings support a behavior-analytic account of this phenomenon. In addition, the review highlights the importance of developing sustained and programmatic lines of research, in which findings are iteratively refined and extended over time (e.g., de Carvalho & de Rose, 2014; Mizael et al., 2016, 2021; Passarelli, Roche et al., 2024b). Given its emphasis on functional relations, contextual control, and derived relational responding, behavior analysis is well positioned to contribute to this area by advancing experimentally grounded analyses of the processes involved.

## Method

A systematic review was conducted in accordance with the Preferred Reporting Items for Systematic Reviews and Meta-Analyses (PRISMA) guidelines (Page et al., 2021). Searches were performed in APA PsycNet across three databases (PsycINFO, PsycARTICLES, and PsycEXTRA). The initial search was conducted between August and September 2025. The search strategy combined keywords related to racial bias (e.g., “racial bias,” “racism,” “racial prejudice”) and applied filters to restrict results to 27 journals historically or conceptually aligned with behavior analysis, using the journal title field within APA PsycNet. The full search strategy is provided in Appendix I. To minimize omissions due to indexing limitations, the search was supplemented by backward snowballing and targeted manual searches in key journals (*Journal of Contextual Behavioral Science, The Psychological Record, Behavior and Social Issues, Behavior Analysis in Practice, Journal of Applied Behavior Analysis, Journal of Experimental Behavior Analysis*). An updated search was conducted in March 2026 using the same procedures, replicating the full search strategy and results across all three databases and 27 target journals to identify additional eligible studies.

Studies were eligible for inclusion if they were empirical (experimental, quasi-experimental, correlational, survey, cross-sectional, or longitudinal), involved human participants, and reported original data on anti-Black bias in particular. This criterion was met when a study measured, analyzed, or intervened on relational or evaluative responding toward Black individuals, Black–white comparisons, or racially relevant stimuli for which Black-specific results were separately reported. To be included, studies also needed to demonstrate a behavioral or contextual-behavioral grounding. This was operationalized as publication in behavior-analytic or CBS-related outlets and/or the use of behavior-analytic or CBS concepts (e.g., reinforcement, relational responding, values, psychological flexibility), even when not experimentally manipulated. Thus, studies employing mainstream methodological approaches (e.g., self-report measures, correlational or group-comparison designs) were included when their conceptual framework or interpretation was grounded in behavior analysis or CBS. The degree of grounding varied across studies, and this variation was considered in the interpretation of findings rather than used as an exclusion criterion.

Only peer-reviewed journal articles were considered. No time constraints were added, that is, all studies were reviewed, regardless of the year in which they were published. Studies were excluded if they were theoretical, conceptual, editorial, or review papers, or if they focused on racial bias in general without providing analyses specific to anti-Black bias. During the full text screening, research based exclusively on cognitive or social-cognitive models without a behavioral interpretation was also excluded (e.g., Aboud et al., 2003; Nesdale & Brown, 2004).

The review process followed four stages:

1. Identification: database searches were conducted using keywords, with filters applied to limit results to predefined journals;
2. Screening: results were exported to Rayyan for systematic review, and titles, abstracts, and keywords were screened using the exclusion criteria;
3. Inclusion, when full-text articles were screened to confirm eligibility based on inclusion criteria; and
4. Extraction, when data were extracted and categorized into three groups:
  - Descriptive, longitudinal studies or surveys measuring the relationship between racial issues and psychological constructs (e.g., inflexibility, depression);
  - Experimental or quasi-experimental studies on measures of racial bias;
  - Experimental or quasi-experimental interventions.

Extracted data included author/year, country of study, study design, participant demographics, measures, and interventions.

It is important to note that a blinded screening procedure was implemented in Rayyan. An independent reviewer, who was not part of the authorship team, conducted a fully blinded review of all records based on inclusion and exclusion criteria. During the title and abstract screening phase, overall agreement between the independent reviewer and the primary screening team was 92%, with agreement on 75 records, 7 conflicts, and 3 records marked as “maybe” by both reviewers. During the full-text screening stage, 100% agreement was achieved. All 10 records (7 conflicts and 3 “maybe”) were excluded at this stage, with reasons for exclusion clearly documented (i.e., not grounded in behavior analysis or not constituting empirical studies about anti-Black bias). This complete agreement at the full-text level further supports the reliability and transparency of the study selection process.

### Methodological Quality and Risk of Bias Assessment

Methodological quality and risk of bias were assessed using a multimethod approach in accordance with PRISMA 2020 (Page et al., 2021). Studies were appraised using design-specific Joanna Briggs Institute (JBI) Critical Appraisal Checklists (Aromataris & Munn, 2020), selected based on methodological purpose: cross-sectional/correlational studies (including measurement-focused designs) using the Analytical Cross-Sectional checklist (Moola et al., 2020), studies involving systematic manipulation of variables using the Quasi-Experimental checklist, and the single randomized controlled trial using the RCT checklist (Tufanaru et al., 2020). In addition, the Single-Case Experimental Design (SCED) Scale (Tate et al., 2020) was applied to one study that met criteria for single-case experimental design with repeated measurement across phases. Studies with small samples that did not meet SCED criteria (e.g., absence of baseline phases or phase-based experimental control) were appraised using the JBI Quasi-Experimental checklist.

Item-level ratings (Yes, No, Unclear, Not Applicable) were used to derive overall quality based on the proportion of applicable criteria met (≥ 70% high, 50%–69% moderate, < 50% low), excluding nonapplicable items. Risk of bias was assessed only for studies involving manipulation of an intervention with comparison between groups, using RoB 2 for the randomized trial (Sterne et al., 2019) and ROBINS-I for nonrandomized studies (Sterne et al., 2022), with overall judgments determined by the most severe domain.

All appraisals were conducted by the first author, with a subset independently assessed by a second blinded reviewer, yielding full agreement. Quality was not used as an exclusion criterion; instead, findings were interpreted in light of both methodological quality and risk of bias, with low-quality or high-risk evidence treated as preliminary.

## Results

**Fig. 1:**
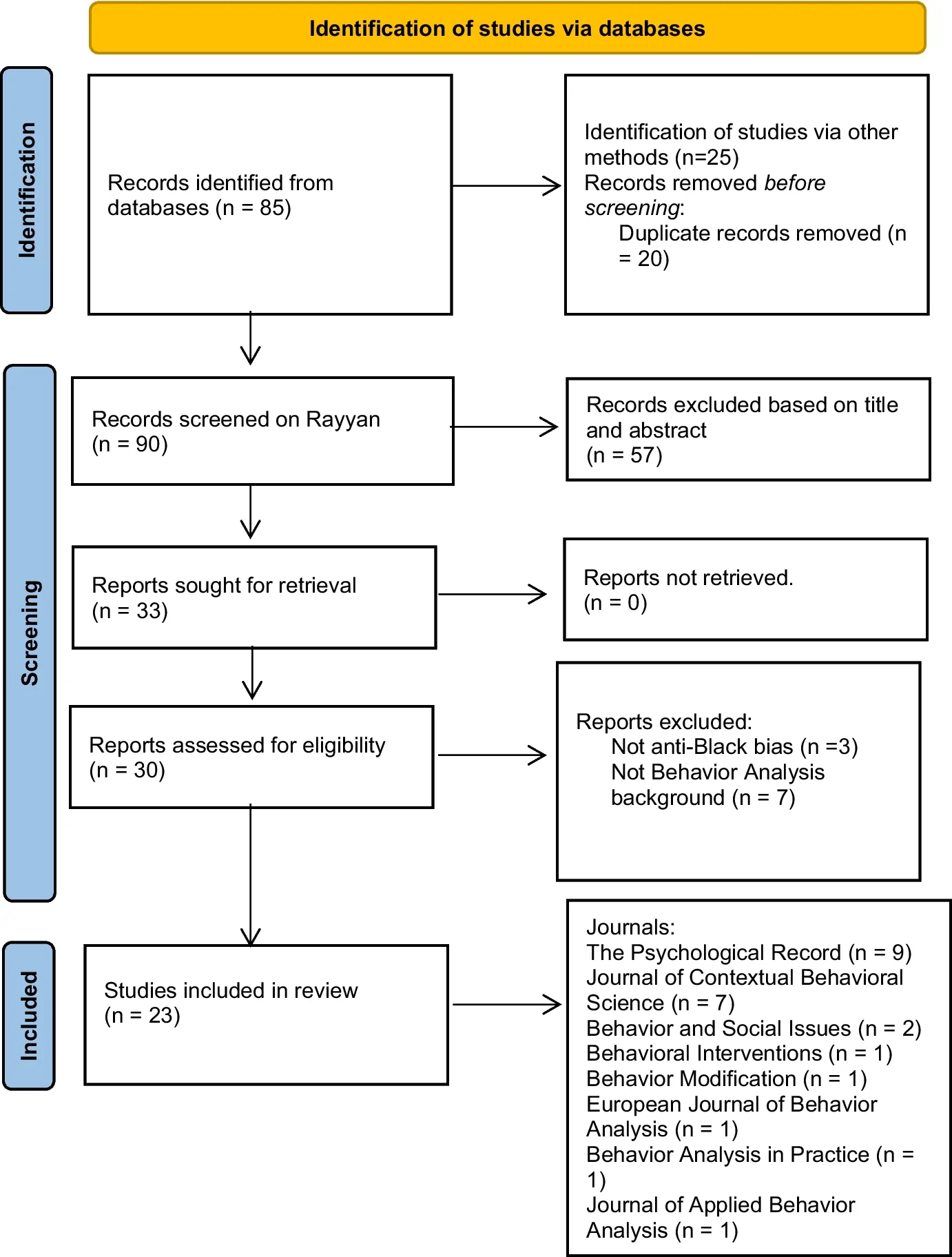
PRISMA Flow Diagram Detailing the Study Selection Process

The search using the keywords, filters, and target journals (see Appendix I) identified 90 articles published between 1974 and 2025. During the screening phase, 57 articles were excluded because they consisted of theoretical papers, opinion pieces, reviews or meta-analyses. The full texts of the remaining 33 empirical papers were then assessed according to the inclusion criteria. Seven studies were excluded because they were not situated within a behavior-analytic background: three because they were from the field of psychobiology, and four due to being from experimental social psychology. Three additional studies were excluded because they did not address anti-Black bias, focusing instead on Asian bias, diversity and inclusion programs, or discrimination toward Indigenous Australian groups. This resulted in a final set of 23 empirical studies. Figure 1 presents the PRISMA flow diagram detailing the study selection process.

These studies span from 1964 to 2025, beginning with a single publication in 1964, 1973 and 2007, followed by three studies between 2010 and 2014 and two in 2015. Publication frequency increased thereafter, with five studies published between 2016 and 2020 and a marked rise from 2022 to 2024, which together accounted for seven studies, the most productive period, distributed as three in 2022, two in 2023, and two in 2024, with an additional study published in 2025 and 2026. Overall, approximately 63% of the studies were published in the last 5 years, indicating a clear upward trend in research activity in this area.

Regarding publication venues, nine studies appeared in *The Psychological Record*, seven in the *Journal of Contextual Behavioral Science* (JCBS), two in *Behavior and Social Issues* (BSI), one in *Behavioral Interventions*, one in the *European Journal of Behavior Analysis* (EJoBA), one in *Behavior Modification*, *Behavior Analysis in Practice* (BAP) and in the *Journal of Applied Behavior Analysis* (JABA).

### Methodological Characteristics of Studies

The included studies varied widely in design and measurement approaches (Table 1). All studies were conducted in three countries: the United States (*n* = 12), Brazil (*n* = 5), and the Republic of Ireland (*n* = 5). Of the 23 studies, 4 employed cross-sectional or correlational designs (Catagnus et al., 2022; Graham et al., 2015; Florez et al., 2019; LoPresti et al., 2022), whereas the remainder used experimental approaches. These included multielement (*n* = 2), within-subject (*n* = 7), mixed within–between (*n* = 4), between-subject (*n* = 1), one ABAB single-case design and one Randomized Controlled Trial (Table 1).

**Table 1:** Methodological Characteristics of the Selected Studies

Authors	Country	Participants	Design	Measures	Intervention
Rice and White (1964)	USA	40 white women (20 college juniors, ages 18–23; 20 noncollege women matched for age).	Between-subjects experimental design	Frequency of stop-light button presses (behavioral interference with opponent); number of times alternate route was taken; verbal impression of opponent (rated by 50 psychologists as accepting, unaccepting, or neutral)	No intervention, participants played a competitive game against a hypothetical White or Negro opponent
Hauserman et al. (1973)	USA	5 Black children (from a classroom of 25 first-grade students	Single-case experimental design (ABAB phases: baseline–prompting–intervention–baseline	Direct behavioral observation of interracial interaction (lunchroom seating and free-play interaction) |	Differential reinforcement of interracial social interaction (teacher prompts + tangible/social reinforcement)
Lillis and Hayes (2007)	USA	32 undergraduate students (13 men, ages 20–37; 21 white, 3 African American, 3 Hispanic, 5 Asian).	Counterbalanced between-groups experimental design	Self-reported behavioral intentions, ACT model-related changes	Group 1: ACT-based session; Group 2: educational lecture on racial differences. A follow-up assessment was conducted 7 days later
Barnes-Holmes et al. (2010a, b)	Republic of Ireland	31 white Irish (15 Male, 16 Woman, *M* = 21 years)	Mixed design (between-subjects × within-subjects)	Discrimination and Diversity Scale (DDS), Modified Modern Racism Scale (MMRS), Likert Scale and the implicit relational procedure assessment (IRAP)	No
West et al. (2013)	USA	14 (2 Male and 12 Female) self-identified as Black Americans	Randomized Controlled Trial	Schedule of Racist Events (SRE), Vividness of Visual Imagery Questionnaire (VVIQ), Positive and Negative Affect Schedule (PANAS), Subjective Units of Distress Scale (SUDS)	Psychoeducation on racism and internalized racial oppression, along with ACT-based strategies
de Carvalho and de Rose (2014)	Brazil	4 children, 7 to 10 years (with racial bias; 1 mixed-race boy, 1 Black girl, 2 white boys)	Within-subject experimental design	Modified Equivalence test and Likert Scale	Equivalence class formation via Matching-to-Sample (MTS) training
Drake et al. (2015)	USA	57 undergraduate psychology students (39 male, *M* = 19.1 years. 22 Black, 22 white, 4 mixed-race, 9 Other)	Within-subjects repeated-measures design	Demographic questionnaire, MMRS, Semantic Differential Scale (SDS), Social Dominance Orientation (SDO)	No
Graham et al. (2015)	USA	57 African American undergraduate and graduate students (*M* = 28.89 years, 74% women)	Correlational, quantitative descriptive design	Schedule of Racist Event—Past Year (SRE-R), Valued Living Questionnaire (VLQ), Depression Anxiety Stress Scales (DASS-21)	No
Mizael et al. (2016)	Brazil	13 children, 8–10 years (7 white, 5 Black, with racial bias)	Within-subject experimental design	Self-Assessment of Manikin (SAM), Modified Equivalence test and IRAP	Equivalence class formation via Matching-to-Sample (MTS) training
Power et al. (2017a)	Republic of Ireland	16 white individuals residing in Ireland (8 Female, *M* = 25 years)	Within-subjects experimental design	IRAP and electroencephalography (EEG) records	No
Power et al. (2017b)	Republic of Ireland	22 Black participants (11 Female, *M* = 22 years)	Mixed design (between-subjects × within-subjects)	IRAP, DDS, SDS and Feeling Thermometer	No
Florez et al. (2019)	USA	253 white college students (*M* = 19.06 years, *SD* = 2.06, 197 females)	Cross-sectional correlational study	Measures of meaning in life, prejudice, values, and obstruction: MLQ-P, PAB, PVQ-RR-ST, VQ-O.	No
Mizael et al., (2021)	Brazil	46 children 8 to 10 years (11 Black, 27 girls) with racial bias demonstrated in pretest	Mixed design (between-groups × within-subjects)	SAM, Modified Equivalence Test and IRAP	Equivalence class formation via Matching-to-Sample (MTS) training. A follow-up assessment was conducted 6 weeks later
Williams et al. (2020)	USA	44 undergraduate students (31 Female, 24 white and 20 Black)	Mixed design (between-groups × within-subjects)	For all participants: PANAS, Allophilia Scale (General/Workshop), Intergroup Anxiety and Avoidance (IAA-6), Satisfaction. For Black participants: Multigroup Ethnic Identity Measure (MEIM-12); for white participants: Color-Blind Racial Attitudes Scale (CoBRAS), Symbolic Racism Scale (SR2K), Color-Conscious Awareness Scale (CCAS-Attitudes and Behaviors).	Group 1: Racial Harmony Workshop (RHW) Group 2: control condition involving a documentary about racism and structured dialogue. A follow-up assessment was conducted 4 weeks later
Banks et al. (2021)	USA	20 Black women aged 18–60 (*M* = 36.26 years, *SD* = 14.92)	Repeated measures within-subjects experimental design	Self-report scales on psychological and racial experiences: DASS-21, Inventory of Race-Related Stress (IROS), Acceptance and Action Questionnaire – II (AAQ-II), Internalized Shame Scale (ISS), Subtle and Blatant Weight Bias Scale (SBWS), SRE, VVIQ, PANAS, SUDS.	Values clarification exercise. A follow-up assessment was conducted 12 weeks later
LoPresti et al. (2022)	USA	202 Black participants (Mean Age = 25.43, 132 female)	Cross-sectional correlational study	Demographic Questionnaire, UMass Boston Comprehensive Race Implicit Bias Measure (UMass Boston Comprehensive), Racial Microaggressions Scale (RMAS), Posttraumatic Stress Disorder Checklist for DSM-5 (PCL-5), Self-Compassion Scale—Short Form (SCSF)	No
Catagnus et al. (2022)	USA	152 participants (117 women; *M* = 37.4 years; 95 white, 53 People of Color (POC), including 14 Black; 4 not disclosed)	Qualitative survey study	Demographic Information, Questionnaire of Emotions Related to Race, Questionnaire Emotions at Work in ABA, Questionnaire of Comfort with Emotions.	No
Romani et al. (2024)	USA	26 adults (21 females, 5 males), aged 27–56, mean 38 years, employed in a children's hospital	Multielement design with baseline and two training conditions (Group 1 and Group 2)	The questionnaires included 15 items each: 5 about microaggressions, 5 nonexamples of microaggressions, and 5 control items.	Differential reinforcement with feedback to help healthcare workers discriminate between examples and nonexamples of racial microaggressions
Belisle et al. (2023)	USA	34 (*M* = 23 years, 31 women, 1 man, 2 nonbinary; *M* = 23 years; 32 white/non-Hispanic, 1 Native American, 1 not disclosed)	Cross-sectional correlational study	Relational Response Measures and Stimuli Characteristics	No
Passarelli, Mizael et al., (2024a), Passarelli, Roche et al., (2024b)	Brazil	9 children (6 girls and 3 boys; *M* = 8.22 years; 5 white, 3 Mixed Race, 1 Black)	Repeated measures within-subjects experimental design	Relational Test, Function Acquisition Speed Test (FAST), Likert Scale, Doll Test.	Equivalence class formation via Matching-to-Sample (MTS) training. A follow-up assessment was conducted 6 weeks later
Passarelli, Mizael et al., (2024a), Passarelli, Roche et al., (2024b)	Brazil	34 children (*M* = 8.6 years; 24 females; Race: 13 white, 7 Black, 14 Mixed-race/Pardo)	Repeated measures within-subjects experimental design	Likert Scale and FAST	No
Suarez et al. (2024)	USA	Experiment 1: 2 Latina women; ages 31 and 39 Experiment 2: 3 adults (42-year-old white male; 38-year-old Black female; 29-year-old Asian female)	Multielement single-case experimental design with no-treatment control	Questionnaire of empathic responding towards different racial groups	Relational training with coordination and distinction frames
Roche et al. (2025)	Republic of Ireland	52 adults (*M* = 22.5 years, 28 Male and 24 Female; Race: 27 white, 25 non-white, mostly mixed-race)	Mixed design (between-subjects × within-subjects)	FAST, DDS, MMRS	No
Passarelli et al. (2026)	Ire Republic of Land	116 white adults (mean age = 23.13 years, *SD* = 3.18; 67 females; 50 undergraduate students + 66 recruited via Prolific)	Between-within subjects randomized experimental design with four conditions	Function Acquisition Speed Test (FAST); Police Officer's Dilemma Task (PODT) Racism Scale (MMRS); Likert scale ratings of Black and White faces	FAST-based prophylactic relational training across four conditions, administered prior to bias assessment

*Note*. “Intervention” refers to procedures designed to modify behavior or psychological processes related to anti-Black bias, including both changes in evaluative responding and reductions in distress associated with racism (e.g., stress or trauma). This also includes experimental manipulations intended to produce measurable change, even when not explicitly labeled as interventions

Self-report measures are clustered into three broad categories. First, measures of psychological functioning, including anxiety, stress, mood, experiential avoidance, and trauma-related variables (Banks et al., 2021; Catagnus et al., 2022; Florez et al., 2019; Graham et al., 2015; LoPresti et al., 2022; West et al., 2013; Williams et al., 2020). Second, measures of racial attitudes and experiences, such as modern racism, colorblindness, ethnic identity, discrimination history, and microaggressions (Banks et al., 2021; Barnes-Holmes et al., 2010a, 2010b; Catagnus et al., 2022; Florez et al., 2019; Lillis & Hayes, 2007; LoPresti et al., 2022; Power et al., 2017a, b; Mizael et al., 2016, 2021; Roche et al., 2025; West et al., 2013; Williams et al., 2020). Third, evaluative measures, including semantic differentials, the Self-Assessment Manikin, Likert-type ratings, feeling thermometers, and equivalence-based evaluations (Barnes-Holmes et al., 2010a, b; Belisle et al., 2023; Mizael et al., 2016, 2021; Passarelli, Mizael et al., 2024a, Passarelli, Roche et al., 2024b; Power et al., 2017a, b; Suárez et al., 2024; Roche et al., 2025; Romani et al., 2024; Williams et al., 2020).

Despite this diversity, most measures were derived from mainstream psychology. Fewer than half of the studies incorporated behavioral or relational assessments, such as the IRAP, FAST, relational tests, or modified equivalence procedures (Barnes-Holmes et al., 2010a, b; Belisle et al., 2023; Mizael et al., 2016, 2021; Power et al., 2017a, b; Passarelli, Mizael et al., 2024a, Passarelli, Roche et al., 2024b; Roche et al., 2025; Suárez et al., 2024). In addition, one study employed a performance-based implicit measure using the Police Officer’s Dilemma Task (Correll et al., 2002) alongside signal detection theory indices to assess racial bias in decision making under uncertainty (Passarelli et al., 2026).

Intervention approaches varied widely, including values clarification, psychoeducation, and procedures derived from acceptance and commitment therapy (ACT), cognitive behavioral therapy (CBT), and functional analytic psychotherapy (FAP), as well as behavior-analytic procedures such as conditional discrimination training (e.g., matching-to-sample) and differential reinforcement with feedback (Banks et al., 2021; LoPresti et al., 2022). These interventions targeted multiple domains, including emotional responding, perspective taking, and evaluative relational responding toward racial stimuli (Romani et al., 2024; Williams et al., 2020). However, relatively few studies assessed maintenance or generalization of effects: only five included follow-up assessments (Lillis & Hayes, 2007; Mizael et al., 2021; Williams et al., 2020; Banks et al., 2021; Passarelli, Mizael et al., 2024a, Passarelli, Roche et al., 2024b), and six evaluated generalization through additional probes or conflict trials (de Carvalho & de Rose, 2014; Mizael et al., 2016, 2021; Passarelli, Mizael et al., 2024a, Passarelli, Roche et al., 2024b; Romani et al., 2024; Suárez et al., 2024).

### Cross-Sectional and Correlational Studies: Description and Methodological Quality

Four cross-sectional or correlational survey studies (Catagnus et al., 2022; Florez et al., 2019; Graham et al., 2015; LoPresti et al., 2022) investigated psychological factors underlying racial attitudes, mental health, values and relational organization underlying prejudiced responding. Methodological quality for these studies was assessed using the JBI Critical Appraisal Checklist for Analytical Cross-Sectional Studies^1^.

Graham et al. (2015) examined whether valued living moderates the relationship between racist experiences and emotional distress among African American adults. Fifty-seven undergraduate and graduate students (*M* = 28.89; 74.2% women) completed measures assessing racist events (SRE-R), valued living (VLQ), and symptoms of depression, anxious arousal, and general anxiety (DASS-21). Valued living was negatively correlated with all symptom clusters. Moderation analyses indicated that racist experiences were associated with greater emotional distress only among participants with lower levels of valued living, suggesting that engagement with personally meaningful life domains may attenuate this association. JBI appraisal using the Checklist for Analytical Cross-Sectional Studies indicated moderate methodological quality overall. All outcome and exposure measures were validated instruments with established psychometric properties in Black samples, and the statistical approach was appropriate for the research question. Inclusion criteria and consideration of potential confounding variables were only partially described, and no information was provided regarding how many individuals were invited to participate or how many declined, preventing assessment of response rate.

Florez et al. (2019) examined whether meaning in life is associated with racial prejudice and whether this relationship is mediated by self-transcendence and psychological inflexibility. The sample consisted of 253 white college students (*M* age = 19.06; 77.9% women) who completed validated measures of meaning in life (MLQ-P), anti-Black prejudice (PAB), self-transcendence values (PVQ-RR-ST), and psychological inflexibility (VQ-O). Meaning in life did not show a significant total effect on prejudice; however, mediation analyses indicated significant indirect effects through both self-transcendence and psychological inflexibility, suggesting that the relationship between meaning and intergroup attitudes operates through value-based and flexibility-related processes. It should be noted that when self-transcendence and psychological flexibility were not accounted for, meaning in life was associated with greater prejudice toward Black individuals. JBI appraisal using the Checklist for Analytical Cross-Sectional Studies indicated moderate methodological quality overall. All instruments demonstrated adequate to good internal consistency in the current sample, and the statistical approach was appropriate for the research question. Inclusion criteria were partially described, and the response rate was not reported. A proportion of cases (15.6%) was excluded during data screening procedures, although details regarding these exclusions were limited.

LoPresti et al. (2022) examined the relationship between racial microaggressions, trauma symptoms, and self-compassion in a sample of 202 Black American adults recruited online and through university networks. Participants completed measures of racial microaggressions (RMAS), trauma symptoms (PCL-5), and self-compassion (SCS-SF). Microaggressions were positively associated with trauma symptoms, with nearly 70% of participants scoring above the clinical cutoff for post-traumatic stress disorder (PTSD), and self-compassion moderated the relationship specifically for invisibility-related microaggressions, reducing but not eliminating their impact. JBI appraisal using the Checklist for Analytical Cross-Sectional Studies indicated moderate methodological quality. Validated instruments with good to excellent internal consistency were used, and gender and income were included as covariates. However, the use of a convenience sample, absence of response rate, incomplete control of confounding, and lack of correction for multiple testing limit the interpretability of the findings.

Catagnus et al. (2022) conducted a quantitative descriptive survey to examine how behavior-analytic practitioners experience emotions in clinical practice and how they perceive emotions related to racism. Using a snowball sampling procedure distributed through email lists and social media, the authors obtained responses from 152 practitioners, predominantly women (117) and primarily white (95), with 53 identifying as people of color, including 14 Black participants. The sample included a broad range of professional certifications: 73 board certified behavior analysts (BCBAs), 34 board certified behavior analysts–doctoral (BCBA-Ds), 18 registered behavior technicians (RBTs), and 3 board certified assistant behavior analysts (BCaBAs). The survey consisted of closed- and open-ended items created by the authors to assess comfort with emotional expression, reactions to emotional situations, and attitudes toward discussing race in professional contexts. Approximately one quarter of the participants (*n* = 39) reported either neutrality or discomfort when discussing issues related to race. Despite this, the vast majority (*n* = 147) agreed that understanding emotions related to racism is essential for competent professional practice, and most participants (*n* = 139) endorsed the view that applied behavior analysis should actively address racism and develop interventions targeting emotions associated with oppression. JBI appraisal using the Checklist for Studies Reporting Prevalence Data indicated low methodological quality. The study relied on nonprobability sampling, did not report a response rate, and used a nonvalidated instrument. No sample size calculation was conducted, confounders were not addressed, and analyses were limited to descriptive statistics, restricting interpretability of the findings.

Methodological quality for cross-sectional and correlational studies is summarized in Table 2, including primary study objectives, JBI scores, and key methodological limitations.

**Table 2:** Methodological Quality of Cross-Sectional and Correlational Studies

Study	Primary Objective	JBI Score	Quality	Key Limitations
Graham et al. (2015)	Anxiety and depressive symptoms associated with racist experiences	5/8	Moderate	Q1 (Sampling and inclusion criteria not clearly described); Q5 (confounders not fully identified); Q6 (no strategies to control confounders)
Florez et al. (2019)	Racial prejudice and its relation to meaning in life and psychological flexibility	5/8	Moderate	Q1 (Sampling and inclusion criteria not clearly described); Q5 (confounders not identified); Q6 (no confounder control strategies)
LoPresti et al. (2022)	Trauma symptoms associated with racial microaggressions	4/8	Moderate	Q1 (Sampling and inclusion criteria not clearly described); Q5 (confounders only partially addressed); Q6 (limited control strategies); Q8 (no correction for multiple comparisons)
Catagnus et al. (2022)	Perceptions of emotions and racism among behavior analysts	3/8	Low	Q1 (Sampling and inclusion criteria not clearly described); Q3 (exposure not measured with validated instrument); Q5 (confounders not identified); Q6 (no control strategies); Q8 (limited statistical analysis)

### Measurement of Anti-Black Bias: Study Description and Quality Appraisal

Most behavioral studies on racial bias have focused on implicit and explicit measurement procedures (except for Rice & White, 1964 and Belisle et al., 2023). The included studies were appraised using the JBI Critical Appraisal Checklists, with the choice of instrument determined by study design. All studies involving systematic manipulation of task parameters (e.g., contextual instructions, response contingencies, or latency criteria), including repeated-measures designs, were appraised using the JBI Checklist for Quasi-Experimental Studies (Nonrandomized Experimental Studies). The only exception was Belisle et al. (2023), which did not involve experimental manipulation and was therefore appraised using the JBI Checklist for Analytical Cross-Sectional Studies. It is important to note that ROBINS-I was not applied because the included studies do not aim to estimate causal effects of interventions or exposures in observational or naturalistic contexts; rather, they examine behavior under controlled experimental conditions, typically using within-subject designs without independent comparison groups, which falls outside the intended scope of ROBINS-I.

Barnes-Holmes et al. (2010a, b), for instance, conducted an experimental nonrandomized study examining the effects of private versus public contexts and response latency on pro-white and anti-Black stereotyping among 31 white Irish participants (15 men, 16 women, *M* = 21 years). The study employed the IRAP to compare performance under blocks requiring pro-white/anti-Black or pro-Black/anti-white responding and manipulated whether participants believed their responses were public or private, as well as the latency criterion required for correct responding. Results showed that the public context did not reduce pro-white bias, and that shorter latency requirements produced stronger stereotype-consistent responding (i.e., anti-Black bias). These findings indicated that immediate relational responding exerted greater control over performance than contextual cues related to public evaluation. JBI for Quasi-Experimental Studies appraisal indicated moderate methodological quality. Strengths include clear temporal ordering, standardized procedures, objective outcome measurement (response latency), transparent attrition criteria, and appropriate statistical analyses. However, no pre-manipulation baseline was obtained, baseline comparability was not formally assessed, IRAP internal reliability was low in the private condition (Cronbach's alpha =.24), and Experiment 2 relied on a nonconcurrent comparison with the public condition from Experiment 1, limiting causal inference.

Drake et al. (2015) conducted a quantitative laboratory study examining the reliability and convergent validity of a race-based IRAP administered twice in succession to an undergraduate sample (*N* = 57). Participants completed self-report measures followed by a practice IRAP and two race IRAPs assessing evaluative relational responses toward Black and white people. Results indicated moderate internal consistency and small-to-moderate test–retest reliability across IRAP administrations. Convergent validity was partially supported, with IRAP effects showing associations with self-report measures of racial attitudes. Trial-type analyses suggested patterns consistent with in-group favoritism rather than explicit out-group derogation. Methodological appraisal using the JBI Checklist for Quasi-Experimental Studies indicated moderate quality. Strengths include standardized behavioral and self-report measures, clear operationalization of outcomes, appropriate statistical analyses, and explicit assessment of reliability and convergent validity. The second IRAP administration was used to assess temporal stability rather than to manipulate an independent variable. Limitations include racially asymmetric attrition, partial nonrandom assignment to groups, absence of clearly defined inclusion criteria, and lack of assessment or control of potential confounders.

Power et al. (2017a) conducted a known-groups study examining racial bias using the IRAP in Black and White participants residing in Ireland (*N* = 34; 16 white, 18 Black after exclusions). The IRAP employed the standard within-subjects block design with consistent (pro-white/anti-Black) and inconsistent (pro-Black/anti-white) contingencies. Results indicated differential patterns of in-group bias, with white participants showing anti-Black bias on the Black-Negative trial type and Black participants showing predominantly positive bias across conditions, alongside evidence of incremental predictive validity over self-report measures. Methodological appraisal using the JBI Checklist for Quasi-Experimental Studies indicated moderate quality. Strengths include standardized measurement procedures, clear operationalization of outcomes, and appropriate statistical analyses. Limitations include nonequivalent group characteristics, lack of demographic matching, incomplete reporting of recruitment and exclusion procedures (particularly for the white sample), and absence of assessment or control of potential confounders such as language proficiency and socioeconomic status. In addition, reliability estimates were inconsistent across groups, with weaker and non-significant effects observed among Black participants.

Power et al. (2017b) conducted a preliminary laboratory study combining the IRAP with concurrent Electroencephalography (EEG) recording in 16 white Irish adults (*M* = 25 years). The behavioral IRAP component employed a within-subjects design contrasting consistent (pro-white/anti-Black) and inconsistent (pro-Black/anti-white) response contingencies and produced robust pro-white/anti-Black responding across all four trial types (ηp^2^ = .92). ERP patterns recorded during IRAP performance indicated greater frontal positivity during stereotype-incongruent blocks, tentatively interpreted as reflecting increased cognitive demands during history-inconsistent relational responding. Methodological appraisal using the JBI Checklist for Quasi-Experimental Studies indicated moderate quality. Although procedures were standardized and statistical analyses were appropriate, confidence in the findings is substantially limited by high attrition in the EEG data, with only nine participants contributing usable signals (approximately 44% attrition), lack of formal reliability assessment for both behavioral and neurophysiological measures, and the exploratory nature of the neurophysiological analyses in the absence of priori hypotheses.

More recent behavior-analytic research has expanded beyond IRAP. Passarelli, Mizael et al. (2024a) conducted a repeated-measures study with 34 children (*M* = 8.6 years), assessing racial bias across three sessions using Modified FAST (Cartwright et al., 2016) and Likert-type evaluative ratings. Across sessions, a subset of children displayed stable implicit anti-Black bias on the FAST, whereas explicit Likert ratings showed no racial preference, highlighting a divergence between implicit and explicit evaluative responding in childhood. JBI appraisal using the Checklist for Quasi-Experimental Studies indicated moderate methodological quality. Strengths include standardized procedures across sessions, explicit assessment of test–retest reliability, appropriate statistical analyses with permutation methods, and absence of attrition. Limitations include the use of a convenience sample, absence of a control group, lack of control for potential confounders, omission of baseline blocks in the modified FAST protocol, and limited generalizability given that effects were observed only in a subset of participants.

Roche et al. (2025) evaluated the sensitivity of the FAST to detect implicit anti-Black racial bias in an online adult sample (*N* = 52 in Experiment 2), using a within-subject design in which participants completed consistent and inconsistent FAST blocks alongside explicit self-report measures. The FAST revealed a significant anti-Black bias at the group level, with effects primarily driven by the white subsample, whereas self-report measures indicated no clear racial preference, replicating the commonly observed divergence between indirect (performance-based) and explicit evaluative responding. Methodological appraisal using the JBI Checklist for Quasi-Experimental Studies indicated moderate quality. Strengths include standardized procedures across participants, appropriate statistical analyses, and transparent reporting of exclusion criteria. Limitations include the absence of a control group, reliance on a relatively novel outcome metric with limited independent validation, and incomplete assessment of the impact of participant exclusions on the results.

Two notable exceptions to the predominant use of implicit measurement paradigms examined anti-Black racial bias without relying on latency-based tasks. Rice and White (1964) conducted a between-subjects experimental study with 40 white women from the southern United States, examining whether racial bias would manifest as competitive and aggressive behavior in a game context. Participants competed against a hypothetical white or Negro opponent, with aggression operationalized as the activation of a stop light to block the opponent’s progress. Results showed significantly more competitive and aggressive responses toward Negro opponents, with no differences as a function of educational level. JBI appraisal using the Checklist for Quasi-Experimental Studies indicated moderate methodological quality due to unclear inclusion criteria, lack of control for confounding variables, absence of pre-/post-measures, and no reported randomization, limiting causal inference and generalizability.

A second notable exception is provided by Belisle et al. (2023), who applied Relational Density Theory to analyze the relational organization underlying prejudiced responding. Participants completed tasks assessing approach–avoidance tendencies and relational clustering among racial stimuli. Results indicated that relational networks involving Black faces clustered with avoidance functions, whereas white-related networks clustered with approach functions. JBI appraisal indicated low methodological quality. The study relied on a small, homogeneous convenience sample, did not define inclusion criteria or control for confounders, and used a primary measure without independent validation and with substantial missing data. Outcomes were derived from aggregate-level multidimensional scaling analyses, which are inherently interpretive and not validated against independent measures. Although model fit was acceptable (stress = 0.09), reliance on aggregated data and absence of individual-level analyses limit interpretability. Therefore, those findings should be interpreted with caution.

Table 3, 4, 5 and 6 presents the methodological quality of behavioral measurements on anti-Black studies, specifying the appraisal tool used, overall scores, and key sources of methodological limitation.

**Table 3:** Methodological Quality of Behavioral Measurement of Anti-Black Bias Studies

Study	Primary Objective	JBI Tool	JBI Score	Quality	Key Limitations
Barnes-Holmes et al. (2010a, b)	Examine effects of assessment context and response latency on racial bias using the IRAP	JBI Quasi-Experimental	6/9	Moderate	Q2 (no control group); Q7 (inconsistent reliability across conditions)
Drake et al. (2015)	Assess reliability and convergent validity of IRAP	JBI Quasi-Experimental	7/9	Moderate	Q2 (no control group); Q3 (non-equivalent groups and partial nonrandom assignment); Q8 (attrition and exclusions uneven across groups)
Power et al. (2017a)	Assess racial bias using IRAP and self-report measures	JBI Quasi-Experimental	5/9	Moderate	Q2 (no control group); Q3 (non-equivalent groups); Q7 (inconsistent reliability across groups); Q8 (unclear completeness of follow-up and exclusions)
Power et al. (2017b)	Explore behavioral and EEG correlates of IRAP performance	JBI Quasi-Experimental	6/9	Moderate	Q2 (no control group); Q7 (reliability not assessed); Q8 (high attrition due to EEG artifacts without impact analysis)
Passarelli, Mizael et al., (2024a), Passarelli, Roche et al., (2024b)	Assess test–retest reliability and implicit bias detection using the FAST	JBI Quasi-Experimental	7/9	Moderate	Q2 (no control group); Q7 (unclear reliability due to modified measurement protocol)
Roche et al. (2025, Exp. 2)	Assess racial bias and FAST sensitivity using within-subject design	JBI Quasi-Experimental	6/9	Moderate	Q2 (no control group); Q7 (novel RFD measure with limited validation); Q8 (unclear completeness of follow-up due to exclusions)
Rice and White (1964)	Assess racial bias through competitive and aggressive behavior in a game context	JBI Quasi-Experimental	6/9	Moderate	Q3 (similarity of treatment conditions unclear); Q5 (no pre-/post-measurements); Q6 (no follow-up)
Belisle et al. (2023)	Relational structure of prejudiced responding	JBI Cross-Sectional	3/8	Low	Q1 (no inclusion criteria); Q3 (measurement not validated); Q5 (confounders not identified); Q6 (no control strategies); Q7 (outcomes not measured in standard/reliable way)

*Note. Q* refers to items from the JBI critical appraisal checklists

**Table 4:** Methodological Quality and Risk of Bias of Stimulus-Equivalence Training with Children

Study	Primary Objective	Appraisal tool	Score	Quality	Key Limitations
Hauserman et al. (1973)	To evaluate interracial interaction and generalization under reinforcement	SCED Scale	7/11	Moderate	Q1 (Participant characteristics not fully detailed); Q7 (Inter-rater reliability was only partially established); Q8 (Independence of assessors not ensured); Q10 (Replication across subjects/settings was limited)
de Carvalho and de Rose (2014)	To examine whether equivalence class training could reorganize negative racial bias in children	JBI Quasi-experimental	4/9	Low	Q2 (No control group); Q7 (unclear measurement reliability); Q8 (no follow-up assessment); Q9 (limited statistical analysis)
Mizael et al. (2016)	To test whether modified training parameters could increase equivalence class formation and reduce racial bias	JBI Quasi-experimental	6/9	Moderate	Q2 (No control group); Q7 (unclear measurement reliability); Q9 (limited statistical analysis)
Mizael et al. (2021)	To evaluate the effects of different training/testing parameters on the formation and maintenance of equivalence classes and racial bias	JBI Quasi-experimental	7/9	Moderate	Q4 (Similar treatment aside from intervention is unclear); Q7 (unclear measurement reliability)
Passarelli, Mizael et al., (2024a), Passarelli, Roche et al., (2024b)	To evaluate the Conflicting Relations Paradigm for reducing racial bias and investigating generalization effects	JBI Quasi-experimental	7/9	Moderate	Q2 (No control group); Q7 (unclear measurement reliability)

*Note. Q* refers to items from the JBI critical appraisal checklists.

**Table 5:** Methodological Quality of Single-Group Interventions

Study	Primary Objective	JBI Tool	JBI Score	Quality	Key Limitations
Banks et al. (2021)	To evaluate a community ACT based intervention on internalized racial oppression	JBI Quasi-experimental	4/9	Moderate	Q2 (no control group); Q3 (baseline comparability unclear); Q4 (similar treatment unclear); Q7 (reliability unclear); Q8 (no follow-up assessment)
Suárez et al. (2024)	Test relational framing effects on empathic responding	JBI Quasi-experimental	5/9	Moderate	Q2 (No control group); Q3 (unclear participant comparability); Q4 (unclear equivalence of care across conditions); Q8 (no follow-up assessment)
Romani et al. (2024)	To evaluate the effects of discrimination training on healthcare professionals’ responses to clinical stimuli	JBI Quasi-experimental	5/9	Moderate	Q2 (Lack of a control group); Q3 (unclear participant comparability); Q4 (unclear equivalence of care); Q8 (no follow-up assessment)

*Note. Q* refers to items from the JBI critical appraisal checklists

**Table 6:** Methodological Quality and Risk of Bias of Between-Group Interventions Targeting Racial Bias and Race-Related Distress

Study	Primary Objective	JBI Tool	JBI Score	Quality	Risk of Bias	Key Limitations
Lillis and Hayes (2007)	Apply acceptance, mindfulness, and values-based processes to reduce racial prejudice	JBI Quasi-experimental	6/9	Moderate	Serious	Q3 (baseline comparability unclear); Q4 (similar treatment unclear); Q7 (self-report outcome) D1—Confounding: Serious; D3—Selection of participants: Serious. D5—Measurement of outcomes: Moderate. D6—Selection of reported results: Moderate
West et al. (2013)	To evaluate whether a brief values-clarification writing exercise buffers racism-related stress among Black American adults.	JBI Quasi-experimental	6/9	Moderate	Serious	Q2—No control group present; Q3—Baseline participants similar (Unclear); Q4—Similar treatment aside from intervention (Unclear).
Williams et al. (2020)	Evaluate ACT-based workshop on racial attitudes	JBI Quasi-experimental	7/9	Moderate	Serious	Q3—Participants similar (Unclear); Q8—Attrition reporting unclear. D1—Confounding: Serious. D2 – Selection of participants: Serious. D6—Measurement of outcomes: Moderate. D7—Selection of reported result: Moderate
West et al. (2013)	Test values intervention on racism-related distress	JBI RCT	9/13	Moderate	Some concerns (RoB2)	Q2—Allocation concealed (Unclear); Q4—No participants blinded; Q5—No providers blinded; Q6—No Outcome assessors blinded. D1—Randomization process: Some concerns. D2—Deviations from intended interventions: Some concerns. D4—Measurement of the outcome: Some concerns. D5—Selection of the reported result: Some concerns.
Passarelli et al. (2026)	Evaluate whether FAST-based relational training reduces racial bias in a simulated police decision-making task	JBI Quasi-experimental	7/9	Moderate	Moderate	Q5 (no baseline/pre-post outcome assessment) and Q8 (no follow-up). D1—Confounding: Moderate; D3—Selection of participants: Moderate; D4—Missing data: Moderate; D5—Measurement of outcomes: Moderate; D6—Selection of reported results: Moderate

*Note. Q* refers to items from the JBI critical appraisal checklists *D* refers to risk-of-bias domains from the ROBINS-I or RoB 2 tools

### Intervention Studies on Anti-Black Bias: Description, Methodological Quality, and Risk of Bias

Exactly half of selected studies (11 out 22) implemented interventions aimed at reducing racial bias or mitigating distress related to racism. Of these, four interventions targeted children and seven targeted adults. To present these findings and methodological appraisal clearly, the intervention literature is organized into three methodological categories. The first group comprises child-focused interventions based on equivalence-based relational training. The second includes adult laboratory studies that manipulate contextual control through relational framing or differential reinforcement. The third group encompasses ACT-based and values-based interventions implemented with adults.

#### Stimulus-Equivalence Training and Differential Reinforcement with Children

Five studies examined interventions with children using behavior-analytic procedures to promote interracial interaction or alter the evaluative functions of racial stimuli. Hauserman et al. (1973) conducted a classroom-based study with five Black children in a predominantly white first-grade setting, evaluating whether differential reinforcement could promote interracial social interaction. Using a phased design (baseline, prompting, reinforcement, withdrawal), the study measured interracial seating and free-play interaction through direct observation. Results showed that reinforcement contingencies increased interracial interaction during lunch and produced generalization to untrained free-play contexts, although effects diminished following withdrawal of reinforcement. SCED appraisal indicated moderate methodological quality (7/11), with strengths including repeated measurement, phase contrast, and evidence of generalization. However, participant characteristics were not fully detailed, interrater reliability was only partially established, assessor independence was not ensured, and replication across independent baselines was limited.

More recent studies (de Carvalho & de Rose, 2014; Mizael et al., 2016, 2021; Passarelli, Mizael et al., 2024a, Passarelli, Roche et al., 2024b) employed stimulus-equivalence procedures to alter the evaluation functions of racial stimuli. All four studies followed the same basic strategy. First, children were screened using a matching-to-sample (MTS) task that reliably identified those who tended to match a negative symbol to Black faces in the presence of competing comparisons, indicating a pre-experimental bias. Next, all studies implemented AB and BC conditional-discrimination training designed to produce derived AC and CA relations without direct training. Typically, A was a positive symbol, B was an abstract stimulus, and C was a Black face, with the aim of transferring the positive evaluative function of A to C through equivalence class formation. Although the basic structure was shared across studies, each introduced procedural variations to enhance class formation, assess maintenance over time, or examine generalization under conflict conditions involving novel Black and white faces. These studies were appraised using the JBI Critical Appraisal Checklist for Quasi-Experimental Studies (Nonrandomized Experimental Studies), given the use of systematic training procedures and repeated measures within participants. ROBINS-I was not applied to de Carvalho and de Rose (2014), Mizael et al. (2016, 2021), and Passarelli, Mizael et al., (2024a), Passarelli, Roche et al., (2024b) because these studies do not include independent comparison groups or estimate between-group causal effects.

de Carvalho and de Rose (2014) implemented AB–BC training with four children selected based on pre-existing negative evaluations of Black faces. All participants acquired the trained relations, but only one showed equivalence-consistent responding between positive stimuli and Black faces, with limited evidence of transfer of functions. The remaining participants showed persistence of pre-experimental response patterns, suggesting that prior relational histories may have influenced performance. JBI appraisal indicated low methodological quality, despite strengths including clear temporal ordering, structured experimental procedures, and objective behavioral outcome measures. However, the absence of a control group, the inability to assess participant comparability, unclear equivalence of care, lack of follow-up assessment, and limited statistical analysis due to the small sample size substantially limit internal validity.

Mizael et al. (2016) expanded the procedure by incorporating simple-to-complex protocol and mixed training, while also comparing simultaneous and delayed MTS. These refinements produced markedly better outcomes: all 13 children formed the targeted classes, and nine maintained the Black–positive relation even when novel white faces were introduced as competing comparisons. Explicit evaluations measured by the Self-Assessment Manikin (SAM) showed that, before training, white faces were rated significantly more positively than Black faces, whereas after training this difference was eliminated. An IRAP administered post-intervention revealed no evidence of implicit racial bias; however, because no IRAP baseline was collected, it was not possible to determine the extent to which the procedure itself influenced implicit responding. Overall, the procedure effectively produced more balanced explicit evaluations, although conclusions about implicit change remain limited. JBI appraisal indicated moderate methodological quality, with strengths including clear experimental control, a systematic training protocol, objective outcome measures, and pre-/post-assessment of bias. However, limitations include the absence of a control group and unclear measurement reliability.

In a subsequent study, Mizael et al. (2021) evaluated three specific parameters derived from the equivalence literature (mixed training, feedback reduction, and simple-to-complex protocol, i.e., symmetry testing), applying each to a different group alongside a control group. Although all groups received the same AB–BC training, the parameters produced distinct effects: 33 of the 46 children formed equivalence classes, with the symmetry group showing the highest yield and the control group showing the lowest. Reductions in bias occurred almost exclusively among children who formed the classes, providing strong support for the functional link between derived relational responding and evaluative change. Maintenance after 6 weeks was moderate but robust among those who had formed classes, with many preserving at least part of the trained and derived relations. JBI appraisal indicated moderate methodological quality, with strengths including clear experimental manipulation, well-defined procedures, objective outcome measures, and inclusion of follow-up assessment. However, limitations include uncertainty regarding whether participants were exposed to similar conditions aside from the intervention, limited reporting on measurement reliability and limited statistical analysis due to the small sample.

Finally, Passarelli, Mizael et al., (2024a), Passarelli, Roche et al., (2024b) employed repeated-measures within-subject design to evaluate a conflicting relations paradigm (CRP) intervention aimed at reducing racial bias in children. Nine participants completed multiple baseline assessments before and after the intervention, as well as a follow-up, using a combination of explicit (relational test, Likert scale, doll test) and implicit (FAST) measures. Results showed a consistent reduction in the racial bias index in the relational test, with elimination of negative associations toward Black faces and reduced valence differences between Black and white faces, although effects were weaker or inconsistent across implicit and generalization measures. JBI appraisal indicated moderate methodological quality, with strengths including repeated baseline assessments, clear temporal sequencing, and the use of multiple outcome measures. However, limitations include the absence of a control group and insufficient reporting of measurement reliability.

#### Reducing Anti-Black Racial Bias and Internalized Racial Oppression: A Single-Group Intervention with Adults

Three studies with adults examined how responding to race-relevant stimuli can be modified through relational processes, reinforcement contingencies, and acceptance- and mindfulness-based strategies. These studies included both participants from the general population and individuals with lived experiences of racial discrimination, targeting both interpersonal responding and internalized racial bias. Across studies, structured interventions were implemented using single-group and within-subject quasi-experimental designs, including multielement arrangements and repeated assessments over time. These studies were appraised using the JBI Critical Appraisal Checklist for Quasi-Experimental Studies, as they involved systematic manipulation of contingencies under controlled conditions to evaluate functional relations between variables.

Banks et al. (2021) implemented a six-session manualized ACT intervention for 20 Black women, combining psychoeducation on racism with exercises in defusion, acceptance, and self-compassion. Pre-/post-assessments using validated measures for African American populations, including the IROS, DASS-21, ISS, and SBW, showed significant reductions in internalized racial oppression, internalized shame, and psychological distress. The program was feasible and well-received, with high retention and clear improvements across targeted processes. JBI appraisal using the Checklist for Quasi-Experimental Studies indicated low methodological quality. Strengths include clear temporal ordering, structured intervention procedures, and consistent application of outcome measures across participants, as well as appropriate statistical analyses. However, limitations include the absence of a control group, lack of clarity regarding baseline comparability, uncertainty as to whether participants were exposed to similar conditions aside from the intervention, reliance on self-report measures with limited evidence of reliability, and the absence of a follow-up assessment.

Suárez et al. (2024) used a multielement arrangement and repeated within-participant comparisons across coordination, distinction, and control conditions. Results showed that coordination cues increased empathic responding toward racial outgroups, whereas distinction cues reduced empathy or produced counterempathic patterns, with replication across participants and across two experiments. JBI appraisal indicated moderate methodological quality, with strengths including clear experimental manipulation, a structured multielement design, repeated measurement across conditions, and replication of effects across participants and experiments. However, limitations include the absence of a control group, unclear participant comparability, uncertainty regarding whether participants were exposed to similar conditions aside from the intervention, and the absence of a follow-up assessment.

Romani et al. (2024) evaluated a differential reinforcement with feedback intervention to train healthcare professionals to discriminate between examples and nonexamples of racial microaggressions. Twenty-six participants completed three sequential assessments (baseline, training for Set 1 or Set 2, and training for the alternate set) within a multielement quasi-experimental design in which participants served as their own controls. Results showed substantial increases in correct discrimination following training, with accuracy improving in a set-specific manner only after exposure to feedback for the corresponding item set, supporting a specific training effect. JBI appraisal indicated moderate methodological quality, with strengths including a structured design, clear temporal sequencing, objective outcome measures, and high interobserver agreement. However, limitations include the absence of a control group, unclear participant comparability, uncertainty regarding whether participants were exposed to similar conditions aside from the intervention, and the absence of a follow-up assessment.

#### Between-Group Interventions Targeting Racial Bias and Race-Related Distress

Five studies employed ACT and related contextual-behavioral interventions to reduce racial bias or alleviate distress associated with experiences of racial bias. These studies involved comparisons between groups and were therefore appraised using design-specific tools: the JBI Checklist for Quasi-Experimental Studies for nonrandomized designs, the JBI Checklist for Randomized Controlled Trials for randomized studies, and risk-of-bias tools aligned with allocation procedures (ROBINS-I for nonrandomized studies and RoB 2 for randomized trials).

Lillis and Hayes (2007) conducted a classroom-based pilot study comparing a single-session ACT intervention with an educational lecture among 32 undergraduate students enrolled in courses on racial issues. Using a counterbalanced sequence of repeated assessments across five time points, the researchers examined changes in awareness of bias, acceptance, cognitive defusion, and prosocial behavioral intentions. The ACT session produced consistent increases in prosocial intentions and ACT-consistent processes immediately after the intervention and at a 1-week follow-up, whereas the educational lecture showed minimal or inconsistent effects. JBI appraisal indicated moderate methodological quality. JBI appraisal indicated moderate methodological quality. Strengths included a structured within-subject time-series design, clear temporal sequencing, and repeated outcome measurement across assessment points. However, limitations included unclear baseline comparability, uncertainty regarding whether participants were exposed to similar conditions aside from the intervention, and reliance on self-report outcome measures, which may be susceptible to social desirability and demand characteristics. Although a follow-up assessment was conducted, its short duration limits conclusions regarding the maintenance of effects over time. ROBINS-I assessment indicated serious risk of bias, primarily due to confounding (Domain 1), including potential carry-over and co-intervention effects inherent to the crossover classroom design, alongside concerns regarding outcome measurement (Domain 5). Findings should therefore be interpreted with caution.

West et al. (2013) conducted a randomized experimental study evaluating whether a brief values-clarification writing exercise could buffer racism-related stress among Black American adults. Fourteen participants listened to an imaginal vignette depicting ambiguous racial discrimination (e.g., receiving worse service without clear justification) before and after completing either a values clarification or a control writing task. Measures of distress and affect, including the SUDS and PANAS, as well as screening instruments such as the SRE and VVIQ, were administered. Results showed large reductions in subjective distress and moderate reductions in negative affect on the values-clarification condition, although statistical significance was not reached potentially due to the small sample. Participants in the control condition, who reflected on their least important values, showed weaker or inconsistent improvements. JBI appraisal using the Checklist for Randomized Controlled Trials indicated moderate methodological quality, reflecting random assignment, use of an active control condition, standardized procedures, and appropriate statistical analyses. However, the study lacked allocation concealment and blinding, and baseline imbalances between groups were observed. Risk of bias was assessed using RoB 2 and judged as some concerns. Although randomization was implemented, the very small sample size and baseline imbalances raise concerns about the randomization process. Additional concerns relate to outcome measurement, as self-report measures were used without blinding. Other domains were judged as low risk, including missing data and adherence to the intended intervention.

Williams et al. (2020) evaluated the Racial Harmony Workshop (RHW), a contextual-behavioral intervention integrating experiential FAP and ACT elements to reduce microaggressions and foster interracial connectedness among Black and white undergraduates. Forty-four participants attended either RHW or an active control condition involving a documentary about racism and structured dialogue. Across multiple measures, including affect, allophilia, intergroup anxiety, racial attitudes, and microaggressive cognitions, the RHW produced stronger gains for white participants, who showed increased positive affect, improved attitudes toward Black individuals, and reductions in microaggressive thoughts and behavioral intentions. Both conditions reduced symbolic and colorblind racism, and Black participants in both groups showed increased ethnic identity. JBI appraisal using the Checklist for Quasi-Experimental Studies indicated moderate methodological quality. Strengths include the presence of a comparator group, clear temporal ordering, validated outcome measures, repeated assessments with follow-up, and appropriate statistical analyses. Limitations include small sample size, potential baseline imbalances, lack of blinding, and absence of explicit strategies to control confounding. ROBINS-I assessment indicated serious risk of bias, primarily due to confounding related to unmeasured participant characteristics, alongside moderate concerns regarding self-report outcome measurement. Other domains were judged as low to moderate risk.

Finally, Passarelli et al. (2026) conducted a between-within subjects’ experiment examining whether FAST-based relational training could prophylactically reduce racial bias in a simulated police decision-making context. A total of 116 white adults residing in Ireland were assigned to one of four conditions: relational flexibility training, stereotype-inconsistent training, stereotype-consistent training, or a no-intervention control. All participants completed the Police Officer's Dilemma Task (PODT), the Modified Modern Racism Scale, and Likert evaluations of novel Black and white faces. Results showed no racial bias in shooting accuracy or signal detection in the flexibility and stereotype-inconsistent conditions, whereas stereotype-consistent and control groups displayed patterns consistent with anti-Black bias. Explicit measures showed no racial bias across conditions. JBI appraisal indicated moderate methodological quality, with limitations including absence of baseline measures, lack of follow-up, and a homogeneous sample. Risk of bias (ROBINS-I) was also judged as moderate, primarily due to lack of participant blinding inherent to the intervention and absence of baseline performance data.

## Discussion

This review reveals that, despite growing conceptual interest in anti-Black racism, empirical investigations within behavior analysis remain limited in both scope and continuity. Over 6 decades, only 23 empirical studies were identified, with the earliest dating to Rice and White (1964) and Hauserman et al. (1973). These pioneering contributions were not followed by sustained lines of inquiry, and empirical activity remained largely dormant for several decades before re-emerging with the ACT-based pilot by Lillis and Hayes (2007). This discontinuity mirrors a broader pattern noted by behavior-analytic scholars: although the field has historically expressed ethical concern about social issues, including racism and inequality, it has often struggled to translate those concerns into sustained empirical research programs (Matsuda et al., 2020; de Souza Silva et al., 2022). Therefore, behavior analysis is only recently beginning to fill a decades-long empirical gap, generating isolated studies rather than a coherent cumulative tradition capable of guiding theory, measurement, and intervention.

It is a paradox that this empirical scarcity stands in contrast to a recent surge in conceptual publications, including special issues devoted to racism and equity. For example, in 2022, *Behavior Analysis in Practice* released a special issue titled “Emergency Series of Publications on Systemic Racism and Police Brutality” (Volume 15, Issue 4), signaling heightened editorial attention to racial injustice (Gingles et al., 2022). Although such contributions have increased visibility and encouraged self-reflection, most emphasized normative discussion rather than experimental analysis. It is important to note that Sidman (1994) warned that conceptual prescriptions unsupported by functional analyses risk weakening the analytic rigor that characterizes behavioral science. A meaningful behavioral engagement with racial justice therefore requires both ethical commitment and a robust empirical foundation.

Across the empirical studies reviewed, the body of work grounded in relational frame theory represents the most substantial methodological and conceptual contribution to understanding racial bias within behavior analysis. The consistent empirical findings from RFT-based research, particularly those utilizing the IRAP and FAST, compel a critical theoretical shift in how racial bias is conceptualized within behavior analysis. The persistence of pro-white/anti-Black responding even under conditions of public scrutiny (Barnes-Holmes et al., 2010a, b) and its temporal stability in children (Passarelli, Mizael et al., 2024a, Passarelli, Roche et al., 2024b; Roche et al., 2025) fundamentally challenge models that attribute bias primarily to explicit attitude or strategic self-presentation. Instead, these data robustly support the interpretation that implicit racial bias operates as a durable, verbally established repertoire of derived relational responding. This repertoire, shaped by community-specific learning histories as evidenced by systematic differences across racial groups (Drake et al., 2015; Power et al., 2017a), functions as a potent source of stimulus control, often overwhelming other contextual cues. The primary contribution here is thus not merely the detection of bias, but the provision of a functional, behavior-analytic account of its fundamental mechanism: bias as a learned pattern of relational framing.

This functional-analytic precision, however, raises critical questions regarding the ecological and predictive validity of these laboratory-based measures. A well-established and persistent limitation in implicit bias research is the modest-to-weak correlation between scores on latency-based tasks and actual discriminatory behavior in naturalistic contexts, a gap that questions their utility for forecasting real-world outcomes (De Houwer, 2019; Greenwald et al., 2015; Oswald et al., 2013, 2015). Consistent with this concern, Corneille and Gawronski (2024) reviewed evidence across domains and concluded that self-report measures tend to outperform implicit measures in predicting socially relevant behavior, including in contexts of racial evaluation, a finding that challenges any straightforward claim that latency-based tools provide superior or uniquely valid assessments of bias. In addition, methodological critiques within behavior analysis have raised concerns regarding the reliability and replicability of IRAP-based research, highlighting issues such as noisy data (Geist et al., 2023; Errasti et al., 2019), variability in effect sizes, and concerns about the sensitivity of the procedure to the attitudes and learning histories it is intended to assess (Hussey & Drake, 2020; Hussey, 2025; Watters et al., 2023).

These critiques do not invalidate the use of implicit measures in racial bias research but underscore the need to treat single-administration findings as preliminary and replication-dependent. What is required is a functional perspective on what these measures capture. From this standpoint, the distinction between implicit and explicit measures is not one of validity, but of controlling variables: whether behavior is governed directly by current environmental contingencies or indirectly by previously established relational networks (De Houwer & Moors, 2012). Implicit measures such as the IRAP and FAST assess relational responding under time pressure, reducing self-presentation and revealing patterns shaped by learning histories (Barnes-Holmes et al., 2010a, b; Watters et al., 2023), whereas self-report measures capture deliberate verbal behavior under conditions that allow reflective and socially mediated responding. Neither is inherently superior, each index behavior under different contextual control. The key question is whether these conditions approximate those under which racial bias occurs in real-world settings, such as contexts involving time pressure, ambiguity, and limited social monitoring (Passarelli, Mizael et al., 2024a). What is needed, therefore, is conceptual clarity about what each tool measures, under which conditions, and for what purpose.

To build a meaningful translational bridge, future research must move beyond the "diagnostic" use of these tools and adopt an experimental analysis of behavior in context. This requires designing studies where implicit relational responding (e.g., IRAP performance) is measured alongside, and as a potential predictor of, functionally defined behavioral outcomes in socially relevant scenarios, such as simulated hiring decisions (Agerström & Rooth, 2011), resource allocation tasks (McConnell & Leibold, 2001), or interactions with confederates (Dovidio et al., 2002). Without such experimental integration that respects the principle of situational specificity (Gawronski, 2019), the claim that these measures capture the "behavioral potential" for discrimination remains an intriguing but insufficiently supported hypothesis.

Intervention studies, although preliminary, show encouraging effects. ACT-based strategies have been used to target values clarification, defusion, and perspective taking, reducing emotional distress associated with racism (West et al., 2013) and internalized racial oppression (Banks et al., 2021), and modifying prejudice-related behavioral intentions (Lillis & Hayes, 2007; Williams et al., 2020). Relational training approaches, including equivalence-based procedures with children (de Carvalho & de Rose, 2014; Mizael et al., 2016, 2021) also demonstrated the capacity to reassign positive functions to Black faces and alter biased relational responding. Although effects varied, particularly in studies involving socially loaded stimuli, these findings support the possibility that carefully arranged contingencies can disrupt pre-experimental relational networks.

A key lesson from the sequence of equivalence-based training studies is not that socially loaded classes are inherently resistant to change, but that the strength of the training procedure is decisive. In the first study, the AB–BC protocol was insufficient to weaken the strong pre-experimental racial networks controlling children’s performances, even though all participants mastered the trained relations (de Carvalho & de Rose, 2014). Across the subsequent experiments, however, methodological refinements such as simple-to-complex protocol, mixed training, controlled feedback, explicit symmetry testing and conflict tests consistently produced the emergence and maintenance of equivalence classes linking Black faces to positive symbols (Mizael et al., 2016, 2021). These convergent findings indicate that pre-experimental frames can be overridden when training includes parameters known to strengthen class formation. Thus, rather than suggesting that equivalence-based procedures are unable to alter socially loaded relations, the developmental trajectory of these studies shows that more robust relational training can reorganize evaluative functions even for culturally entrenched stimuli.

Adult intervention studies, in turn, demonstrate that functionally distinct mechanisms can each produce meaningful change in racial bias-related outcomes. ACT-based approaches modified the contextual control that relational networks exert over behavior through defusion, acceptance, and values clarification, reducing distress and increasing prosocial behavioral intentions (Banks et al., 2021; Lillis & Hayes, 2007; West et al., 2013; Williams et al., 2020). Relational procedures, in contrast, targeted the networks themselves, producing more functionally specific changes in empathic responding and in behavioral discrimination (Romani et al., 2024; Suárez et al., 2024). These approaches are better understood as complementary than competing, and a cumulative research agenda would benefit from studies that examine how they interact in producing durable reductions in discriminatory behavior.

Approximately 65% of the studies identified in this review were published within the past 5 years, indicating a sharp increase in behavior-analytic engagement with racial issues. This recent growth appears closely tied to the broader sociocultural shift following the 2020 murder of George Floyd, which generated unprecedented global mobilization around anti-Black racism and placed renewed pressure on scientific fields to address racial injustice (see Esquierdo-Leal & Houmanfar, 2021; Ghezzi et al., 2022). In the same year, Matsuda et al. (2020) published a conceptual article arguing that behavior analysis has both the tools and the ethical responsibility to study racism as a behavioral phenomenon shaped by cultural contingencies. Soon after, *Behavior Analysis in Practice* launched a special issue dedicated to diversity, equity, and inclusion (2022), explicitly inviting empirical and conceptual work on racism, bias, and social justice. These events collectively created a clear contextual occasion for increased research productivity in the area.

However, this growth reveals a concerning pattern of conceptual segregation: empirical studies have been concentrated in a small number of behavior-analytic and contextual behavioral science journals, primarily *The Psychological Record*, *JCBS*, and *Behavior and Social Issues*, although remaining largely absent from flagship experimental and applied outlets. Although one early study appeared in the *Journal of Applied Behavior Analysis* (Hauserman et al., 1973), no subsequent work on anti-Black bias has been published in that journal, and JEAB has yet to feature any empirical study in this area. This distribution reflects more than a thematic affinity; it signals that the analysis of racism is still marginal to the field's core experimental and applied agendas. For this area to evolve from a reactive trend to a cumulative science, active endorsement from the field's principal archives is required. Following *Behavior Analysis in Practice*'s lead, journals such as JEAB and JABA could catalyze this maturation by explicitly soliciting work on the behavioral science of racism, through special sections, targeted calls, or dedicated editorship. This institutional commitment would not only elevate the topic's scientific standing but would critically subject it to the highest standards of experimental design, ensuring it becomes a sustained program of inquiry rather than a transient subfield.

A pervasive methodological limitation across the reviewed studies is the reliance on Western, educated, industrialized, rich, and democratic (WEIRD) samples, a pattern not unique to behavior analysis but widely recognized in psychological science more broadly (Henrich et al., 2010). Cross-sectional and correlational studies received low to moderate methodological quality ratings, with shared limitations including incomplete specification of inclusion criteria, absence of response rate reporting, and limited control for confounding variables, constraints that restrict conclusions to theoretically plausible associations rather than functional or causal relationships (Catagnus et al., 2022; Florez et al., 2019; Graham et al., 2015; LoPresti et al., 2022). Measurement studies were uniformly rated as moderate quality, with recurrent limitations including the absence of independent control groups, inconsistent reliability estimates across conditions, and reliance on convenience samples (Barnes-Holmes et al., 2010a, b; Belisle et al., 2023; Drake et al., 2015; Power et al., 2017a, b), indicating that conclusions about bias detection are generally sound within controlled conditions but that comparisons across studies and inferences about real-world behavior remain constrained. Intervention studies showed the most heterogeneous risk profile, with single-group designs rated as low to moderate quality and between-group studies frequently associated with moderate to serious risk of bias, primarily due to confounding, absence of randomization, and reliance on self-report outcomes susceptible to demand characteristics (Banks et al., 2021; Lillis & Hayes, 2007; Williams et al., 2020), with only Passarelli et al. (2026) receiving a moderate overall ROBINS-I judgment. These quality and risk of bias patterns are best understood not as disqualifying the reviewed evidence, but as marking the field's current developmental stage and informing how findings across each study category should be interpreted and extended.

It is important to note that within behavior analysis, the question of sample representativeness carries a particular nuance. The field has historically been criticized for relying on small samples, sometimes involving a single organism, leading to claims that behavior-analytic research lacked generality. In response, foundational work emphasized that the validity of behavioral science does not depend on statistical representativeness, but on the orderly replication of functional relations across individuals and conditions, a position that contributed to the establishment of the *Journal of the Experimental Analysis of Behavior* (JEAB; Sidman, 1994; Skinner, 1956). On the other hand, most studies in this review did not employ traditional single-subject arrangements, instead adopting group-based, correlational, or cross-sectional designs (e.g., Banks et al., 2021; Catagnus et al., 2022; Florez et al., 2019; Graham et al., 2015; Lillis & Hayes, 2007; LoPresti et al., 2022; Williams et al., 2020), reflecting a form of methodological borrowing from other research traditions (Critchfield, 2011; Mace & Critchfield, 2010).

This “methodological borrowing” carries both risks and opportunities. The risk lies in conceptual drift: when group-based or correlational designs are adopted without sufficient behavior-analytic reframing, the resulting analyses tend to describe topographical covariations between constructs rather than functional relations between behavior and its controlling variables. In these cases, the language of behavior analysis may be present, but the logic of functional analysis is not, and explanations risk sliding toward mentalistic accounts or cause-and-effect inferences that obscure the environmental variables responsible for the observed patterns (Baer et al., 1968; Skinner, 1953 ). At the same time, standardized appraisal tools prioritize criteria aligned with group-based designs and may not fully capture the analytic aims of studies that are conceptually grounded in behavior analysis but methodologically drawn from other research traditions. In addition, because these studies draw on such methods while being interpreted within a behavior-analytic framework, they are often evaluated using criteria (e.g., hypothesis testing and statistical control) that do not fully align with their analytic goals, which may contribute to lower methodological ratings.

The opportunity, however, is equally relevant. When interpreted within a behavior-analytic framework, cross-sectional and correlational findings can help identify socially relevant contextual variables and patterns of relational responding that may inform subsequent functional analyses. In this sense, methodological borrowing can serve as a complementary strategy for guiding the analysis of behavior under more controlled conditions (Critchfield, 2011; Mace & Critchfield, 2010). This gradient does not warrant post-hoc exclusion but does call for analytic precision when drawing conclusions about the specific contributions of behavior analysis as a distinct scientific tradition to the study of racial bias.

Beyond the design-specific limitations discussed above, additional limitations constrain the cumulative strength of the evidence. Most studies did not incorporate systematic replication across settings or populations, and longitudinal follow-up was uncommon. Only a subset of interventions included follow-up assessments (Banks et al., 2021; Lillis & Hayes, 2007; Mizael et al., 2021; Passarelli, Roche et al., 2024b; Williams et al., 2020). Even among these, the longest follow-up period did not exceed 12 weeks, limiting conclusions about the persistence and functional stability of behavior change over time. Furthermore, many interventions lacked tests of generalization or maintenance, restricting inferences about whether experimental effects extend to new stimuli, contexts, or response classes.

None of the included studies reported preregistration of procedures or analytic plans. Although preregistration is not yet standard practice in behavior-analytic research, it would offer clear benefits to the field. Because the experimental analysis of behavior depends on transparent identification of independent and dependent variables, stable baselines, precise procedural descriptions, and replication of functional relations, preregistration could strengthen the field by reducing analytic flexibility, clarifying the distinction between confirmatory and exploratory analyses, and increasing the credibility and reproducibility of reported findings. Together, these limitations underscore the need for greater procedural transparency and cumulative methodological rigor in future investigations.

A conceptual limitation across studies was the inconsistent definition of racial bias, with many using terms such as “attitudes,” “prejudice,” or “racism” without translating them into behavioral processes. Although recent work has begun to articulate functional definitions grounded in relational responding and stimulus transformation (Mizael & de Rose, 2017; Passarelli et al., 2023; Passarelli, Mizael et al., 2024a, Passarelli, Roche et al., 2024b), as well as alternative formulations such as relational density theory (Belisle et al., 2023), these advances have yet to be systematically adopted across empirical studies. This disconnect may reflect the challenges of translating conceptually elaborated behavior-analytic accounts into experimentally tractable research, as well as continued reliance on methodological approaches that do not fully align with functional-analytic frameworks. Addressing this gap will likely require the development of programmatic lines of research that more directly integrate conceptual advances with empirical investigation.

A final consideration involves the scope of the review itself, in particular its exclusive focus on anti-Black bias within behavior-analytic journals. Although this delimitation necessarily excludes important work on other forms of racism (e.g., anti-Indigenous, anti-Asian) and research published in broader interdisciplinary outlets, it was a necessary methodological constraint. It served as a focused lens to critically assess whether the field has developed a sustained, programmatic research agenda on a specific form of racial bias. The finding of profound scarcity and discontinuity is therefore not an artifact of a narrow search; rather, it is the diagnostic outcome. Had a robust, cumulative literature on anti-Black bias existed within the field's core outlets, this review would have captured it. Its absence underscores a fundamental challenge: for behavior analysis to contribute meaningfully to the science of equity, it must move beyond sporadic, context-reactive studies and commit to building structured, continuous research programs, focused on anti-Blackness and other systemic prejudices. The inability to find such a program within a deliberately targeted search is arguably the review's most significant, and troubling, finding.

### Call-to-Action: Future Directions

The empirical gaps charted in this review underscore the need to build a more systematic, programmatic research agenda on racial bias within behavior analysis. One possible barrier to the development of such an agenda may be structural. As discussed by Faber et al. (2023), processes such as covert exclusionary practices, power-hoarding dynamics, and an “illusion of inclusion” within contextual behavioral science and related organizations may contribute to the marginalization of Black scholars and communities. Such conditions may limit the development of sustained research programs by constraining the range of perspectives that inform research questions and intervention design.

Even when studies yield null or weak outcomes, these results should not be taken as definitive evidence that anti-racist programs are ineffective or that bias is immutable (see Lai et al., 2016; Faber et al., 2023; Forscher et al., 2019). From a behavior-analytic perspective, such outcomes may instead indicate that the conditions arranged were not sufficient to produce robust or generalized effects, highlighting the need to examine additional variables and complementary strategies that may be required to influence complex forms of socially mediated behavior (Sidman, 1994).

A useful illustration of this process is provided by a line of studies initiated by de Carvalho and de Rose (2014), extended by Mizael et al. (2016, 2021), and more recently by Passarelli, Mizael et al. (2024a), Passarelli, Roche et al. (2024b). Initial findings showing limited or null effects were followed by systematic refinements in procedural parameters, leading to more consistent and replicable reductions in negative evaluations toward Black faces using equivalence-based training. This line of work illustrates how early outcomes can inform the development of more effective arrangements, supporting the gradual establishment of reliable effects.

It is important to note that in other domains of behavior analysis, such as autism intervention, initial failures are typically followed by continued refinement, replication, and investment (Morris, 2009). By comparison, the relative scarcity of sustained research programs on racism may reflect broader contextual factors, including limited funding streams and shifting social priorities. This pattern may be interpreted as suggesting that issues related to racism have not yet been fully established as a stable focus of disciplinary investment (Critchfield & Reed, 2017; Fawcett, 1991).

Advancing this line of work may therefore require greater emphasis on systematic and parametric replication, rather than isolated pilot studies, to identify conditions under which biased responding can be reliably modified. Although implicit measures such as the IRAP and FAST provide precise analyses of relational responding under controlled conditions, their relevance to real-world behavior may depend on the extent to which they are embedded within ecologically valid contexts. Bridging this gap may involve extending experimental arrangements to naturalistic settings, incorporating socially meaningful stimuli, and measuring overt behavior under conditions that approximate real-world contingencies.

From this perspective, the development of a sustained research agenda on racial bias may be closely tied to broader structural and disciplinary priorities. The current pattern of limited and intermittent empirical work may reflect not only scientific challenges but also the degree to which this topic is institutionally supported. Expanding this area of research may involve increasing dedicated funding, creating editorial spaces that explicitly value this work, and supporting training pathways that broaden participation in the field.

Overall, the patterns observed in this review suggest the need for a more cumulative, rigorous, and conceptually coherent behavior-analytic program of research on racial bias. This effort will also require the more consistent integration of functionally precise definitions within empirical research, ensuring that constructs are explicitly linked to behavioral processes. Future work may benefit from the use of representative samples, preregistered protocols, transparent reporting practices, and behavioral measures that capture functionally relevant actions. Interventions may also need to extend beyond brief laboratory tasks to examine behavior in socially consequential contexts. If behavior analysis is to contribute meaningfully to issues of racial equity, progress will likely depend on the development of a sustained empirical science capable of analyzing and modifying the contingencies that maintain racial inequality.

## Supplementary Information

Below is the link to the electronic supplementary material.

Supplementary file1 (DOCX 36 KB)

## Data Availability

All articles included in this review were accessed legally through institutional subscriptions via Periodicos CAPES. All materials used in this review derive from publicly available peer-reviewed empirical publications. Screening files and data extraction sheets can be made available by the authors upon reasonable request.

## Footnotes

1 This approach was selected because the included studies were designed to examine associations between variables rather than to estimate causal effects of an exposure on an outcome. Therefore, risk-of-bias tools developed for causal inference (e.g., ROBINS-E) were not applied, as their use requires an explicit causal framework that was not the aim of these studies.
