# Supplementary material for: A Systematic Review of Empirical Data from Behavior Analysis on Anti-Black Bias
Source: Perspect Behav Sci. 2026 Jun 9;49(3):523–68. doi: 10.1007/s40614-026-00506-3 (PMC13562460; doi:10.1007/s40614-026-00506-3)
Supplement: Supplementary file 1 [file 40614_2026_506_MOESM1_ESM.docx]

**Appendix I – Search Strategy**

(Any Field: "Racial Bias" OR Any Field: Racism OR Any Field: Prejudice) AND Journal Title: {Journal of the Experimental Analysis of Behavior} OR {Corrective & Social Psychiatry & Journal of Applied Behavior Therapy} OR {Journal of Applied Behavior} OR {Journal of Applied Behavior Analysis} OR {Journal of Applied Behavioral Science} OR {Behavior and Social Issues} OR {Behavior Analysis in Practice} OR {European Journal of Behavior Analysis} OR {Behavior Analysis & Social Action} OR {Behavior Analysis: Research and Practice} OR {Perspectives on Behavior Science} OR {Behavior Modification} OR {Behavioral Interventions} OR {Learning & Behavior} OR {Behavioral and Brain Sciences} OR {Journal of Behavioral Education} OR {Journal of Organizational Behavior Management} OR {Analysis of Verbal Behavior} OR {Journal of Contextual Behavioral Science} OR {International Journal of Behavioral Consultation and Therapy} OR {International Journal of Behavioral Development} OR {International Journal of Behavioral Medicine} OR {International Journal of Behavioral Sciences} OR {Behavioural Processes} OR {Behavioural Analysis & Modification} OR {European Journal of Behavioural Analysis & Modification} OR {International Behavioural Scientist} on Databases APA PsycINFO AND APA PsycArticles AND PsycExtra.
